# Dual treatment with a fixed ratio of glucagon and insulin increases the therapeutic window of insulin in diabetic rats

**DOI:** 10.14814/phy2.13657

**Published:** 2018-03-29

**Authors:** Christina Pedersen, Stephan D. Bouman, Trine Porsgaard, Mette M. Rosenkilde, Nikolaj K. Roed

**Affiliations:** ^1^ Department of GLP‐1 & T2D Biology Novo Nordisk A/S Maaloev Denmark; ^2^ Faculty of Health and Medical Sciences Department of Biomedical Sciences University of Copenhagen Copenhagen Denmark; ^3^ Department of Insulin Pharmacology Novo Nordisk A/S Maaloev Denmark

**Keywords:** Diabetes, glucagon, hypoglycemia, insulin

## Abstract

The current available insulin therapies decrease blood glucose but are associated with the risk of developing hypoglycemia. Glucagon is a counter regulatory hormone and we hypothesize that a fixed ratio of insulin and a long‐acting glucagon‐analogue can reduce the risk of hypoglycemia. To define an appropriate ratio we tested two fixed glucagon doses (3.5 and 10 nmol/kg) in combination with increasing doses of insulin in diabetic rats. We observed a plateau in blood glucose at 15.2 mmol/L with 10 nmol/kg of the glucagon‐analogue. The mechanism behind this plateau, protecting against hypoglycemia, was investigated by measuring the glucose output, cAMP production, and hormone binding in primary rat hepatocytes. While glucose output could contribute to the observed plateau in blood glucose, cAMP response or hormone binding did not explain the observation. Though such plateau indicated decreased risk of hypoglycemia a full normalization of blood glucose was still needed. Based on the data obtained with 3.5 nmol/kg of the glucagon‐analogue, a 1:23 (glucagon‐analogue:insulin) ratio was chosen and a dose‐response was performed in diabetic rats. At low doses (≤20 nmol/kg), insulin and the 1:23 ratio showed similar efficacy of lowering blood glucose. Interestingly, the insulin‐dose resulting in hypoglycemia was increased from 40 nmol/kg insulin alone to 160 nmol/kg insulin in the 1:23 ratio. Analysis of the liver glycogen content at the end of the experiment showed that the highest dose in the 1:23 ratio almost emptied the liver from glycogen. Thus, liver glycogen is essential for the protective effect of glucagon in hypoglycemia.

## Introduction

Insulin and glucagon are counteracting hormones in regulation of blood glucose (Cryer 1993). Insulin lowers blood glucose, while glucagon increases blood glucose. For many years, insulin has been a vital part of diabetes treatment. However, although insulin therapies decrease blood glucose, they are also associated with the risk of developing hypoglycemia. American Diabetes Association and European Association for the Study of Diabetes define clinically significant hypoglycemia as blood glucose levels below 3.0 mmol/L (Group IHS, 2016). Hypoglycemia results in a range of symptoms progressing from sweating and hunger to cognitive dysfunction and seizures (Cryer et al. 2003). Furthermore, hypoglycemia has been associated with increased risk of cardiovascular events and mortality (Ismail‐Beigi et al. 2010; Khunti et al. 2015). Thus, the fear of hypoglycemia is a major limiting factor in the glycemic control of diabetes (Cryer 2002). In order to reduce the hypoglycemic risk, some patients maintain their blood glucose concentrations above the recommended levels (Anderbro et al. 2015). The resulting poor metabolic control is associated with an increased risk of future diabetic complications. Therefore, hypoglycemia needs to be addressed for better management and treatment of patients with diabetes.

Usually counter regulatory responses, such as secretion of glucagon and adrenaline, oppose the effects of insulin when the blood glucose concentration falls below 3.9 mmol/L (Sprague and Arbeláez 2011). However, it is well known that these counter regulatory responses to hypoglycemia are gradually lost in both type 1 diabetes and advanced type 2 diabetes (Cryer et al. 2003). On account of these defects, insulin therapy alone is unlikely to achieve ideal control and safety. In order to improve the glycemic control a dual hormone therapy may be needed. Today, glucagon is only used to treat severe insulin‐induced hypoglycemia in diabetes. The closed‐loop artificial pancreas, imitating normal physiology by administering either insulin or glucagon, demonstrates the feasibility of a safe blood glucose regulation in individuals with type 1 diabetes (Ward et al. 2008; El‐khatib et al. 2010; Haidar et al. 2013). Thus, several studies have shown a beneficial effect of glucagon in reducing the risk of hypoglycemia in insulin therapies. Opposed to the artificial pancreas, which administers either insulin or glucagon depending on the blood glucose level, we investigated whether coadministration of a fixed ratio of the two hormones could reduce the risk of hypoglycemia when treating diabetic rats.

## Materials and Methods

### Compounds and formulations

NPH insulin (Novo Nordisk A/S) was used for daily treatment in diabetic rats. For in vivo experiments, human insulin (Novo Nordisk A/S), and a long‐acting glucagon‐analogue (Novo Nordisk A/S) were used. Both were formulated in 5 mmol/L phosphate, 140 mmol/L NaCl, 70 ppm polysorbate‐20, pH 7.4. For in vitro experiments human insulin (Novo Nordisk internal reference solution) and native glucagon (Novo Nordisk A/S) dissolved in 20% H_2_O and 80% DMSO were used.

### Rat hepatocytes

Hepatocytes from ad libitum‐fed male Sprague‐Dawley rats (∼200 g) were isolated in situ by a two‐step perfusion technique (Seglen 1976; Andersen et al. 1999). Cell viability, determined with a NucleoCounter (Chemometec), was consistently greater than 85%. Hepatocytes were diluted to a cell density of 0.5 × 10^6^ cells/mL in basal medium (Medium 199 (Gibco #41150, Thermo Fisher Scientific) supplemented with 100 nmol/L dexamethasone, 1% penicillin streptomycin (Gibco #15140‐122, Thermo Fisher Scientific) and 1 nmol/L insulin with 4% FBS (Gibco #26140, Thermo Fisher Scientific) and plated in collagen‐coated plates (400 *μ*L in 24 well plates for glycogenolysis and 100 *μ*L in 96 well plates for cAMP and binding assay). To remove dead cells, the medium was changed to basal medium with 0.1% FBS 3 h after initial plating. The competition binding and cAMP measurements were performed on the next day. For glycogenolysis experiments, the medium was changed after 24 h to basal medium supplemented with 0.1% FBS, 9.5 mmol/L glucose and 9 nmol/L insulin, and experiments were then performed on day 3.

### Glycogenolysis

The hepatocytes were washed twice in PBS without CaCl_2_ and MgCl_2_ (Gibco #14190‐094, Thermo Fisher Scientific) prior to preincubation for 30 min at 37°C in medium 199 without glucose (Gibco #041‐94001M, Thermo Fisher Scientific). They were then stimulated for 1 h at 37°C with increasing ligand (glucagon and/or human insulin, Novo Nordisk A/S) concentrations (0–1 *μ*mol/L). Glycogenolysis was measured as glucose released into the medium using a glucose oxidase kit (BioVision #K606‐100). These data were plotted as dose‐response curves and normalized to the maximum response generated from the stimulation with glucagon.

### cAMP production

The hepatocytes were washed twice in HBSS buffer (HBSS with CaCl_2_ and MgCl_2_ (Gibco #14025, Thermo Fisher Scientific) supplemented with 10 mmol/L HEPES (Sigma‐Aldrich #H3375)) prior to preincubation for 30 min at 37°C in HBSS buffer in addition of 0.1% pluronic (Gibco #24040‐032, Thermo Fisher Scientific) and 500 *μ*mol/L IBMX (Sigma‐Aldrich #I7018‐100MG, dissolved in DMSO). The hepatocytes were then stimulated for 30 min at 37°C with increasing ligand (glucagon and/or human insulin, Novo Nordisk A/S) concentrations (0–1 *μ*mol/L). After aspiration of the ligands, the cells were lysed immediately in cold lysis buffer for 10 min. The lysates were transferred to a 384 well optiplate and detecting reagents provided in the kit (cAMP dynamic 2, Cisbio Bioassays, Codolet, France) were added. After incubation in the dark at room temperature for 2 h, the signal of fluorescence resonance energy transfer was read using the Envision 2104 Multilable Reader. The concentration of cAMP produced was interpolated from a standard curve (prepared according to the kit). These data were plotted as dose‐response curves and normalized to the maximum response generated from stimulation with glucagon.

### Whole‐cell radioligand binding assay

The hepatocytes were washed twice in HBSS buffer prior to preincubation for 30 min on ice in assay buffer (HBSS buffer supplemented with 0.1% pluronic and 1% ovalbumin (Sigma‐Aldrich #A5503)). The hepatocytes were incubated overnight at 4°C with 60 pmol/L ^125^I‐glucagon (Novo Nordisk A/S) or ^125^I‐insulin (Novo Nordisk A/S) and increasing concentrations of unlabeled glucagon or insulin. Unbound ligand was washed off, and the cells were lysed in 0.1 M NaOH prior to addition of MicroScint‐40. The radioactivity was measured as cpm in a TopCount NXT gamma counter (PerkinElmer Life Sciences).

### Experimental animals

Male Sprague‐Dawley rats (300 g.) were obtained from Taconic, Lille Skensved, Denmark. All animals were group housed with a 12‐h dark/light cycle and had free access to food and water. All rats had at least 1 week of acclimatization before induction of diabetes. The experiments were approved by the Danish Animal Experiment Inspectorate, under Danish Ministry of Justice, and are in accordance with the Danish Animal Experimentations Act. The studies were performed in the Laboratory Animal Facility at Novo Nordisk A/S, Maaloev, Denmark.

### Streptozotocin‐induced diabetes

On the day of treatment, preweighed STZ (Sigma‐Aldrich) was dissolved in 0.1 mol/L cold citric acid buffer (65 mg/mL, pH 4.5). Diabetes was induced in isoflurane‐sedated rats by injection of 65 mg/kg STZ. The following days, regular inspections were performed and from day 3 the rats were dosed subcutaneously with NPH insulin (Novo Nordisk A/S, 600 *μ*mol/L, 3U/rat/day). Experiments were performed between 2 and 5 weeks after STZ treatment. Only rats with basal blood glucose levels above 15 mmol/L were included.

### Study designs

All experiments were performed in nonfasted diabetic rats. The rats were randomly assigned to dosing groups. In one set of experiments the rats were dosed subcutaneously (1 mL/kg) with insulin alone (0–40 nmol/kg) or insulin (up to 160 nmol/kg) in combination with fixed doses of glucagon (10 nmol/kg or 3.5 nmol/kg). In another set of experiments, the rats were dosed subcutaneously with insulin alone (0–40 nmol/kg) or with a fixed 1:23 (glucagon‐analogue:insulin) ratio. Blood glucose was measured repeatedly for 6 h. Rats with symptomatic hypoglycemia received oral glucose and were excluded from data analysis. At the end of one of the experiments with fixed 1:23 ratio, the rats were anesthetized (5% isoflurane) and liver samples were collected, freeze clamped in liquid nitrogen, and stored at −80°C until analysis of glycogen content.

### Blood glucose measurements

To assess the maximum blood glucose lowering effect, blood from the tail vein (10 *μ*L) was collected in capillary tubes and transferred to 500 *μ*L system‐solution. Blood glucose levels were analyzed using the glucose oxidase method at a Biosen glucose analyzer (EKF Diagnostics, Barleben, Germany) according to manufacturer's instructions.

### Determination of liver glycogen content

Approximately 30–40 mg of frozen liver tissue was homogenized in 1 mL sodium acetate buffer (0.15 mol/L, pH 4.9) supplemented with Triton X‐100 (0.75%). The tissue homogenate was heated to 100°C for 2 min, cooled on ice and mixed thoroughly. The homogenate was then divided in two; one part for measurement of free glucose concentration and a second part for measurement of total glucose concentration. For measurement of free glucose, the homogenate was centrifuged at 9000 G for 10 min before the supernatant was collected and stored at −20°C. For measurement of total glucose concentration, the homogenate was incubated with amyloglucosidase (Sigma‐Aldrich A1602) overnight at room temperature before centrifugation and collection of the supernatant. The total and free glucose concentrations were measured using the Cobas 6000 analyzer (Roche). The glycogen content in the tissue samples was then calculated by subtracting the free glucose from the total glucose concentrations.

### Statistics

GraphPad Prism was used to fit and analyze the data. Multiple *t*‐tests were used to compare two mean values. Prism computed an unpaired *t*‐test for each row, followed by multiple comparisons using the Holm‐Sidak method. For comparison of three or more mean values, a one‐way analysis of variance (ANOVA) followed by Tukey's multiple comparisons test was used. A *P* value below 0.05 reflects a statistically significant difference.

## Results

### Insulin in combination with fixed doses of glucagon in diabetic rats

**Figure 1:**
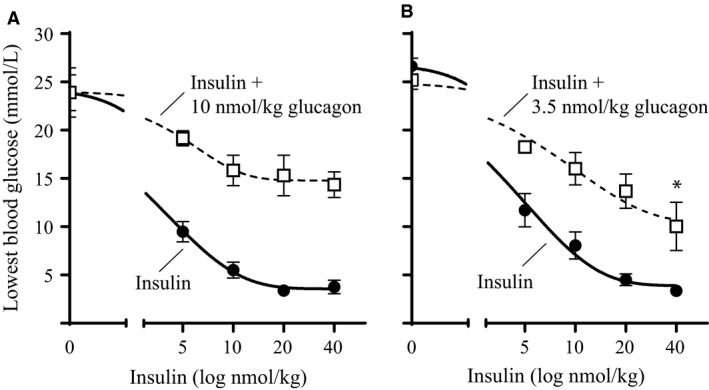
Insulin in combination with fixed doses of glucagon in diabetic rats. The lowest^1^ blood glucose value (mmol/L) obtained during the 6‐h experiments plotted as function of insulin dose (log nmol/kg). (A) Dose‐response of insulin ± 10 nmol/kg glucagon‐analogue. (B) Dose‐response of insulin ± 3.5 nmol/kg glucagon‐analogue. The solid black lines represent the insulin dose‐response curves (0–40 nmol/kg). The dotted black lines represent similar doses of insulin in the presence of the glucagon‐analogue. All data are expressed as means ± SEM; *n* = 6 per group. * indicate significant difference between “5 nmol/kg insulin + 3.5 nmol/kg glucagon‐analogue” and “40 nmol/kg insulin + 3.5 nmol/kg glucagon‐analogue” (*P* = 0.013), (analyzed using one‐way ANOVA followed by Tukey's multiple comparisons test). ^1^The points corresponding to 0 nmol/kg insulin are basal blood glucose levels from the “vehicle group” and the “glucagon‐analogue group”, respectively.

To define an appropriate glucagon:insulin ratio for future experiments two glucagon doses (10 and 3.5 nmol/kg) in combination with increasing doses of insulin (0–40 nmol/kg) were tested in diabetic rats. The effect was evaluated by looking at the lowest blood glucose values measured during the 6‐h experiments. In two individual experiments (Fig. 1A and B), insulin alone lowered blood glucose levels in a dose‐dependent manner from 23.9 ± 1.9 mmol/L to 3.8 ± 0.7 mmol/L and from 26.6 ± 0.8 to 3.4 ± 0.6 mmol/L, respectively. The long‐acting glucagon‐analogue did not result in a significant change in blood glucose on its own (data not shown). However, in combination with insulin 10 nmol/kg of the glucagon‐analogue decreased the glucose lowering effect of insulin resulting in a plateau at 15.2 ± 1.7 mmol/L for insulin doses of 10, 20, and 40 nmol/kg (Fig. 1A). In combination with 10 nmol/kg of the glucagon‐analogue, there was no significant difference in the blood glucose lowering effect of 5 nmol/kg insulin and 40 nmol/kg insulin. A similar plateau in blood glucose was not seen with the 3.5 nmol/kg dose of the glucagon‐analogue in combination with insulin (Fig. 1B). In combination with 3.5 nmol/kg of the glucagon‐analogue, 40 nmol/kg insulin lowered the blood glucose significantly compared to 5 nmol/kg insulin (*P* = 0.013).

### Glycogenolysis, radioligand binding, and cAMP in rat hepatocytes

**Figure 2:**
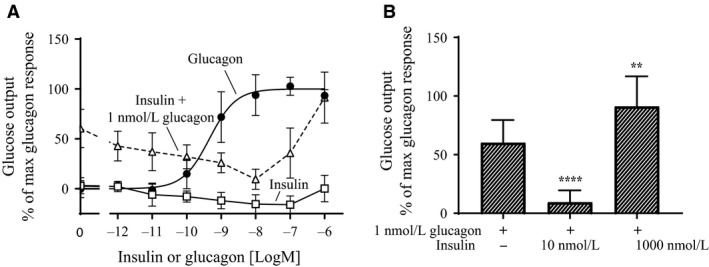
Glycogenolysis in primary rat hepatocytes. (A) Glucose output from the hepatocytes after stimulation with increasing concentrations of glucagon (•), insulin (□) and insulin + 1 nmol/L glucagon (Δ). All data were normalized to max. glucagon response and plotted as a function of ligand concentration [logM]. Data represent means ± SD from three (*d*/*r* insulin) or four (*d*/*r* glucagon and *d*/*r* insulin + 1 nmol/L glucagon) independent experiments carried out in triplicates and normalized to max. glucagon response in each experiment. (B) Normalized glucose output from the hepatocytes after stimulation with “1 nmol/L glucagon”, “1 nmol/L glucagon + 10 nmol/L insulin” and “1 nmol/L glucagon + 1000 nmol/L insulin”. ****indicate significant difference between “1 nmol/L glucagon” and “1 nmol/L glucagon + 10 nmol/L insulin” (*P* < 0.0001) and **indicate significant difference between “1 nmol/L glucagon” and “1 nmol/L glucagon + 1000 nmol/L insulin” (*P* = 0.0012) (analyzed using one‐way ANOVA followed by Tukey's multiple comparisons test).

To understand the mechanism of the observed plateau in blood glucose we measured glycogenolysis in primary rat hepatocytes. As expected, glucagon stimulated glucose output from the hepatocytes in a dose‐dependent manner with a logEC_50_ of −9.34 logM (~0.5 nmol/L) (Fig. 2A). Insulin alone did not have any significant effect on the glucose output. However, in combination with 1 nmol/L glucagon insulin showed a biphasic response. At low concentrations (<10 nmol/L) insulin inhibited glucose output from the hepatocytes, but at higher concentrations it stimulated glucose output (Fig. 2A). As illustrated in Figure 2B, 10 nmol/L insulin significantly inhibited the glucagon‐induced glucose output (*P* < 0.0001), whereas 1000 nmol/L insulin significantly stimulated the glucagon‐induced glucose output (*P* = 0.0012). Even though, this challenges the general view of the opposing interplay between insulin and glucagon, it could potentially explain the observed plateau in blood glucose (Fig. 1A).

**Figure 3:**
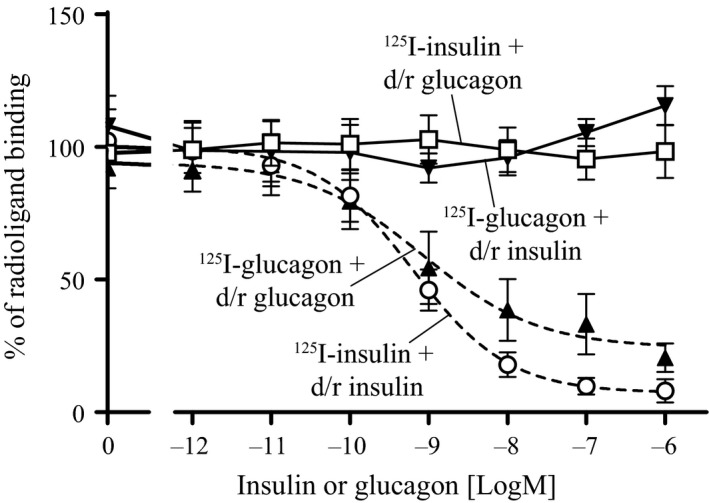
Whole cell radioligand binding on primary rat hepatocytes. Whole cell binding of ^125^I‐glucagon or ^125^I‐insulin after incubation with a dose‐response (*d*/*r*) of either glucagon or insulin. All data were normalized to the respective max. binding of ^125^I‐radioligand (closed symbols denotes ^125^I‐glucagon, and open symbols denotes ^125^I‐insulin) and plotted as a function of ligand concentration [logM]. Homologous binding curves (dotted curves) are included as positive controls. Data represent means ± SD from three independent experiments carried out in triplicates and normalized to max. binding of ^125^I‐radioligand in each experiment.

Whole‐cell radioligand binding on primary rat hepatocytes was used to clarify whether the interplay between insulin and glucagon was happening at the level of receptor binding. As expected from the molecular differences in the receptors of these two hormones, insulin neither displaced nor enhanced binding of glucagon to the glucagon receptor. Similarly, glucagon neither displaced nor enhanced binding of insulin to the insulin receptor (Fig. 3). This indicates that insulin does not bind to the glucagon receptor and vice versa. Consequently, the enhanced glycogenolysis observed at high concentrations of insulin was not mediated by an increased binding of glucagon to its receptor in the presence of insulin.

**Figure 4:**
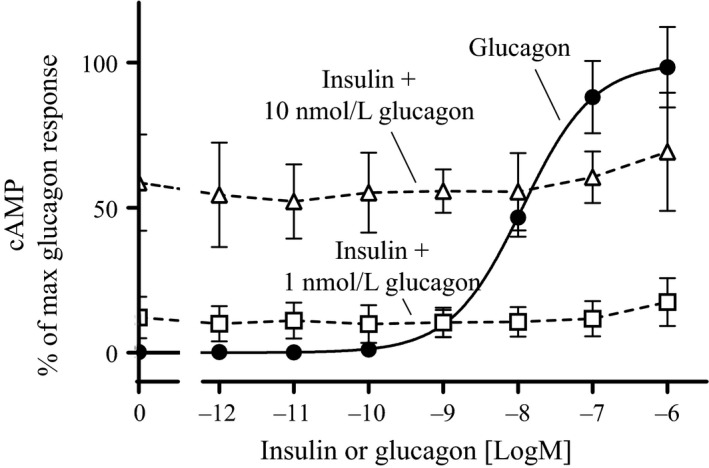
cAMP production in primary rat hepatocytes. cAMP production after stimulation with increasing concentrations of glucagon (•), insulin + 1 nmol/L glucagon (□) and insulin + 10 nmol/L glucagon (Δ). All data were normalized to max. glucagon response and plotted as a function of ligand concentration [logM]. Data represent means ± SD from two independent experiments carried out in triplicates and normalized to max. glucagon response in each experiment.

Glucagon is known to stimulate glucose output through a cAMP‐dependent mechanism (Christophe 1995; Jiang and Zhang 2003). Thus, to investigate the interplay at a downstream level, the cAMP response was measured in primary rat hepatocytes. Glucagon stimulated cAMP production in a dose‐dependent manner with a logEC_50_ of −7.94 logM (~10 nmol/L) (Fig. 4). Insulin in combination with either 1 nmol/L or 10 nmol/L glucagon resulted in cAMP production corresponding to the effect of the fixed glucagon doses. Insulin did not have any additional effect on the cAMP production (Fig. 4). Hence, the observed plateau in blood glucose and the stimulatory effect of insulin on the glucagon‐induced glucose output occurs through a cAMP‐independent mechanism.

### A fixed ratio of glucagon and insulin in diabetic rats

**Figure 5:**
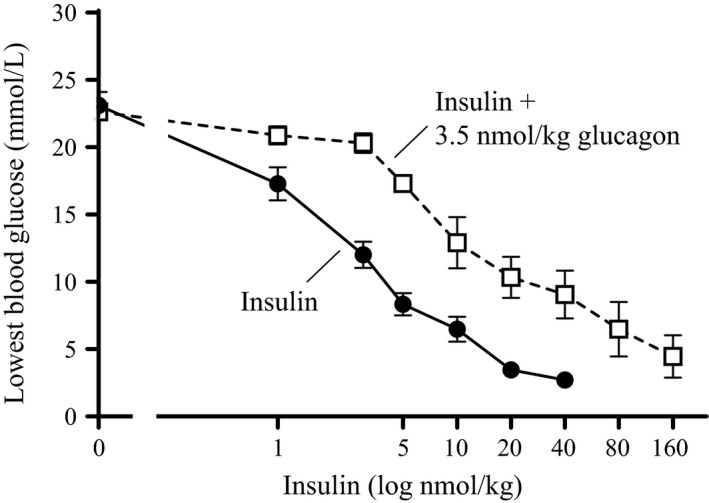
Defining the optimal fixed ratio of insulin and glucagon in diabetic rats. The lowest^1^ blood glucose levels (mM) obtained during the 6‐h experiment plotted as function of insulin dose (log nmol/kg). • represents increasing doses of insulin (0–40 nmol/kg). □ represents increasing doses of insulin (0–160 nmol/kg) + 3.5 nmol/kg glucagon‐analogue. All data are expressed as means ± SEM; *n* = 6–7 per group. ^1^The points corresponding to 0 nmol/kg insulin are basal blood glucose levels from the “vehicle group” and the “3.5 nmol/kg glucagon‐analogue group”, respectively.

**Figure 6:**
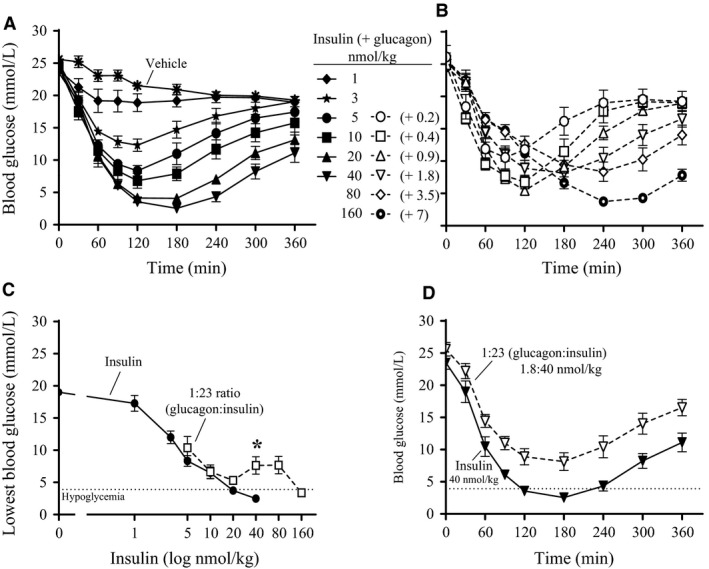
Insulin compared to a fixed 1:23 (glucagon:insulin) ratio in diabetic rats. Blood glucose plotted as function of time. At *t* = 0 min the animals were dosed subcutaneously with (A) insulin alone (0–40 nmol/kg) or (B) a fixed 1:23 (glucagon:insulin) ratio (0.2–7 nmol/kg glucagon in combination with 5–160 nmol/kg insulin). Blood glucose was measured in tail tip blood for 6 h. (C) The lowest blood glucose levels (mmol/L) obtained during the 6‐h experiment plotted as function of insulin dose (log nmol/kg). • represents increasing doses of insulin alone. □ represents increasing doses of the 1:23 ratio. *indicate significant difference (*P* < 0.05) between 40 nmol/kg insulin and the corresponding 1:23 ratio (1.8 nmol/kg glucagon: 40 nmol/kg insulin) (Analyzed using unpaired *t*‐tests for each row, followed by multiple comparisons using the Holm‐Sidak method). (D) Direct comparison of blood glucose as function of time for 40 nmol/kg insulin and the corresponding 1:23 ratio (1.8 nmol/kg glucagon:40 nmol/kg insulin). Data are means of two individual experiments ± SEM; *n* = 6–18 per group.

Driven by the protective effect of dual treatment with insulin and glucagon on hypoglycemia (Fig. 1), we went on to determine the optimal ratio between the two hormones. An optimal ratio would provide full normalization of blood glucose with additional benefit of reduced hypoglycemic risk. In order to do this, we increased the insulin dose (up to 160 nmol/kg) in combination with 3.5 nmol/kg of the glucagon‐analogue. Again, insulin alone (0–40 nmol/kg) lowered blood glucose levels in a dose‐dependent manner from 23.1 ± 1.0 to 2.7 ± 0.4 mmol/L (Fig. 5). The addition of 3.5 nmol/kg of the glucagon‐analogue resulted in a right‐shift of the insulin dose‐response curve and a subsequent decreased potency of insulin. An insulin‐dose of 80 nmol/kg in combination with 3.5 nmol/kg of the glucagon‐analogue only lowered blood glucose to 6.5 ±2.0 mmol/L, a level that was reached by 10 nmol/kg insulin in the absence of the glucagon‐analogue (Fig. 5). This ratio corresponding to 1:23 (glucagon‐analogue:insulin) was chosen and a dose‐response experiment was performed in diabetic rats. An expected dose‐response was observed for 0–40 nmol/kg insulin alone (Fig. 6A). In the 1:23 ratio the insulin dose administered ranged from 5 to 160 nmol/kg, and a shift in lowest blood glucose value towards later time points was observed (Fig. 6B). There was no significant difference between the maximum blood glucose lowering effect of 5, 10, and 20 nmol/kg insulin alone and the 1:23 ratio (Fig. 6C). This indicates similar potency of insulin alone and the 1:23 ratio in the dose‐range normalizing blood glucose. However, with a higher dose of insulin (40 nmol/kg) in combination with the glucagon‐analogue (1.8 nmol/kg) a significant (*P* < 0.05) increase in blood glucose was observed (Fig. 6C and D). This indicates a protective effect of glucagon against hypoglycemia. The insulin‐dose resulting in hypoglycemia was increased four times: from 40 nmol/kg insulin alone to 160 nmol/kg insulin in the presence of the glucagon‐analogue (Fig. 6C).

**Figure 7:**
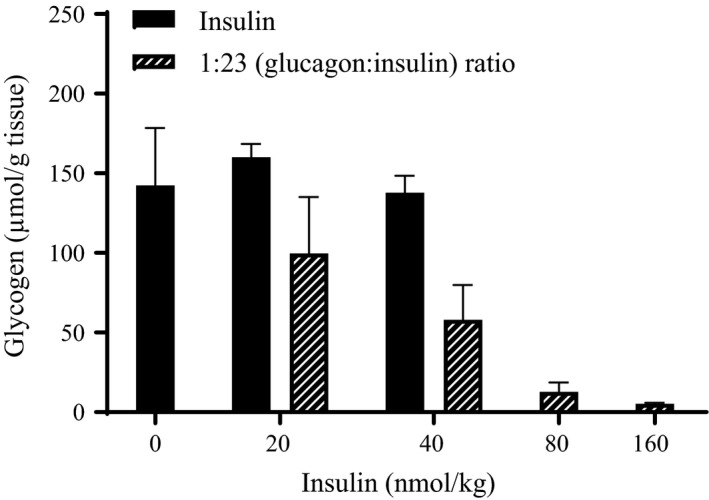
Liver glycogen content. Liver glycogen content (*μ*mol/g tissue) 6 h after s.c. dosing with insulin (black bars) or the 1:23 (glucagon:insulin) ratio (shaded bars) in diabetic rats. All data are expressed as means ± SEM; *n* = 2–6 per group.

After the experiment liver samples were collected and the amount of glycogen was determined. Glucagon stimulated glycogenolysis as indicated by reduced glycogen stores in the liver with increasing doses of glucagon in the 1:23 ratio (Fig. 7). The amount of glycogen was reduced from 142 ± 36 *μ*mol/g in the vehicle‐treated group to 5.2 ± 0.8 *μ*mol/g with the highest dose in the 1:23 ratio (7 nmol/kg glucagon + 160 nmol/kg insulin).

## Discussion

We hypothesized that coadministration of glucagon and insulin could reduce the risk of hypoglycemia when treating diabetes. In rats, when trying to define an ideal ratio we observed a plateau in blood glucose after administering a fixed dose of a long‐acting glucagon‐analogue in combination with increasing doses of insulin (Fig. 1A). Extending this curve would probably confirm the observed plateau. However, since the plateau was around 15 mmol/L and we aimed for a treatment that could normalize blood glucose, we found it redundant and unethical to waste animals in an extended experiment. Nevertheless, we found it interesting and investigated the mechanism behind the plateau by measuring glycogenolysis in primary rat hepatocytes. The results indicated that insulin in combination with glucagon had a biphasic response (Fig. 2A); at concentrations below 10 nmol/L insulin inhibited glucose output from the hepatocytes opposed to insulin concentrations above 10 nmol/L which stimulated glucose output from the hepatocytes. Despite this being in contrast to the general understanding, where insulin and glucagon are only considered as counteracting hormones, it could potentially explain the observed plateau in blood glucose. Other investigations of the interplay between insulin and glucagon have focused on the effects at more physiological concentrations (Cherrington et al. 1976; Cherrington 1999). The basal insulin concentration in plasma is individual, but typically around 60 pmol/L in fasted state (Keith 2010). Though, it oscillates in line with food intake up to 800 pmol/L (König et al. 2012). The action of the two hormones at supra‐physiological concentrations has been studied to a very limited extent. An insulin concentration of 10 nmol/L has been shown to suppress glucagon‐stimulated glucose production in men (Bomboy et al. 1977). Similar results have been obtained when looking at liver perfusion in vitro (Mackrell and Sokal 1969). However, in our case the stimulation of glucose output from the hepatocytes was only observed at insulin concentrations above 10 nmol/L and thus never reported before. Such a high concentration of insulin may seem unrealistic and therefore probably not investigated or overlooked by others. Importantly, our studies show that the conventional understanding of the counteracting regulation of blood glucose by insulin and glucagon appears to count only for physiological, but not pharmacological, concentrations of the two hormones. At supra‐physiological concentrations, a biphasic curve appears for insulin with enhanced effect of glucagon‐induced hepatic glucose output (Fig. 2A); a phenomenon that was not explained by receptor cross‐reactivity (Fig. 3) or amplification of glucagon‐induced cAMP production (Fig. 4). In contrast to the glucagon receptor, which is a G protein‐coupled receptor, the insulin receptor activates G protein‐independent pathways, and hence do not per se elicit a cAMP response. Accordingly we find that the stimulatory effect of insulin on the glucagon‐induced glucose output occurs through a cAMP‐independent mechanism. Yet the mechanism remains to be identified. We have looked at other signaling targets involving phosphorylation of cAMP response element‐binding protein and activation of phospholipase C, but those did not give any glucagon response in primary rat hepatocytes (data not shown). Despite the lacking mechanistic understanding we find the phenomenon interesting and the data important to share. Other techniques (e.g., phospho‐proteomics) are more suitable for identification of pathways behind the mechanism. That would, however, require much more resources and was out of the scope in this study.

Our goal was to find a fixed ratio of glucagon and insulin which could combine the ability to normalize blood glucose with reduced risk of hypoglycemia in diabetic rats. Increasing doses of insulin in combination with 3.5 nmol/kg of the glucagon‐analogue could normalize blood glucose. Therefore, these data were used to define the fixed 1:23 ratio of glucagon and insulin. At low doses (≤20 nmol/kg insulin), the 1:23 ratio showed similar efficacy of lowering blood glucose as insulin alone. Thus, there was no glucagon‐effect with low doses of the 1:23 ratio (<0.9 nmol/kg glucagon‐analogue). It is essential to avoid significantly increased circulating insulin levels after dual treatment with insulin and glucagon compared to insulin treatment alone. Since insulin is also a growth factor and is associated with weight gain an increased insulin dose is unwanted for diabetic patients. In this study, the glucagon‐effect appeared right before hypoglycemia occurred. This might be explained by the glucose‐dependent actions of glucagon. During euglycemia, it has been demonstrated that insulin is a potent inhibitor of glucagon's action on the liver (Steiner et al. 1990). During hypoglycemia, glucagon has been shown to significantly stimulate hepatic glucose production, despite a 35‐fold increase in insulin (Dobbins et al. 1991). Furthermore, a direct comparison of glucagon‐action during euglycemia and hypoglycemia also concludes that the hepatic sensitivity to glucagon is increased during insulin‐induced hypoglycemia (Rivera et al. 2010). Overall indicating that, in the hypoglycemic state, glucagon is able to overcome the inhibitory effect of insulin on hepatic glucose production. The insulin‐dose resulting in hypoglycemia was increased from 40 nmol/kg insulin alone to 160 nmol/kg insulin in the 1:23 ratio. These data indicate that, compared with insulin alone, the therapeutic window was increased four times when administering a fixed 1:23 ratio of glucagon and insulin. Importantly, analysis of the liver glycogen content at the end of the experiment showed that the highest dose in the 1:23 ratio (7 nmol/kg glucagon‐analogue + 160 nmol/kg insulin) almost emptied the glycogen stores from the liver (Fig. 7). This explains why glucagon was not able to protect against hypoglycemia with this dose in the 1:23 ratio. Previously, a study in dogs has shown a correlation between the hepatic glucose output in response to insulin‐induced hypoglycemia and glycogen content in the liver (Winnick et al. 2016). Also, the glucagon effect during hypoglycemia has been shown to be dependent on the preceding carbohydrate intake (Ranjan et al. 2017). For 1 week, patients with type 1 diabetes had either a high‐carbohydrate diet or a low‐carbohydrate diet. The glucagon‐effect was evaluated by administration of 100 *μ*g glucagon after mild insulin‐induced hypoglycaemia (plasma glucose at 3.9 mmol/L). Even though, glycogen stores were not measured it is very likely that low carbohydrate intake reduced the glycogen stores and thus caused the reduced glycemic response to glucagon. Taken together, this indicates a higher risk of developing hypoglycemia when less glycogen is available. We do not intend to treat with a dose emptying the liver from glycogen. We aim for a treatment that provides protection against hypoglycemia if overdosed. Another important note is that our experiments were performed without food present. Access to food will indeed increase the liver glycogen content. More studies are needed to investigate the glucagon‐effect after multiple dosing and in the presence of food. In addition, it would be relevant to consider the effect of other counterregulatory hormones for the complete understanding of the protective mechanisms during hypoglycemia.

In summary, these data support the hypothesis that a fixed ratio of insulin and a long‐acting glucagon‐analogue can reduce the risk of hypoglycemia. We have shown that a 1:23 ratio of the glucagon‐analogue and insulin increases the therapeutic window of insulin in diabetic rats. Importantly, this was obtained without compromising the potency of insulin in the dose‐range normalizing blood glucose. If this can be translated to humans it is very likely to improve the glycemic control for the diabetic patients. With less fear of hypoglycemia, the patients will be able to adhere to tighter glycemic control and thereby reduce the risk of developing diabetic complications later in life. However, it is important to bear in mind the preliminary nature of these findings as well as the limitations of the study to fully clarify the clinical benefits. For instance, the liver glycogen content was found to be essential for the protective effect of glucagon in hypoglycemia. Additional studies are needed to assess whether glycogen content is a limiting factor after multiple dosing with the fixed ratio of glucagon and insulin. Moreover, this study was performed on STZ‐induced diabetic rats, which is considered as a type 1 diabetic animal model. It will be relevant to study the fixed ratio of glucagon and insulin in a type 2 diabetic animal model where a higher insulin dose is needed because of more insulin resistance. Additionally, a type 2 diabetic animal model can be used to address potential anti‐obesity effects of glucagon (Parker et al. 2013).

## Conflict of Interest

C. P., S. D. B., T. P., and N. K. R. are employed by Novo Nordisk A/S.
